# Exploring new uses for existing drugs: innovative mechanisms to fund independent clinical research

**DOI:** 10.1186/s13063-021-05273-x

**Published:** 2021-05-04

**Authors:** Ciska Verbaanderd, Ilse Rooman, Isabelle Huys

**Affiliations:** 1 Department of Pharmaceutical and Pharmacological Sciences, KU Leuven, Leuven, Belgium; 2 Anticancer Fund, Strombeek-Bever, Belgium; 3 Oncology Research Centre, Vrije Universiteit Brussel, Brussels, Belgium

**Keywords:** Drug repurposing, Clinical research, Research funding, Public-private partnerships, Crowdfunding, Social impact bonds

## Abstract

**Background:**

Finding new therapeutic uses for existing medicines could lead to safe, affordable and timely new treatment options for patients with high medical needs. However, due to a lack of economic incentives, pharmaceutical developers are rarely interested to invest in research with approved medicines, especially when they are out of basic patent or regulatory protection. Consequently, potential new uses for these medicines are mainly studied in independent clinical trials initiated and led by researchers from academia, research institutes, or collaborative groups. Yet, additional financial support is needed to conduct expensive phase III clinical trials to confirm the results from exploratory research.

**Methods:**

In this study, scientific and grey literature was searched to identify and evaluate new mechanisms for funding clinical trials with repurposed medicines. Semi-structured interviews were conducted with 16 European stakeholders with expertise in clinical research, funding mechanisms and/or drug repurposing between November 2018 and February 2019 to consider the future perspectives of applying new funding mechanisms.

**Results:**

Traditional grant funding awarded by government and philanthropic organisations or companies is well known and widely implemented in all research fields. In contrast, only little research has focused on the application potential of newer mechanisms to fund independent clinical research, such as social impact bonds, crowdfunding or public-private partnerships. Interviewees stated that there is a substantial need for additional financial support in health research, especially in areas where there is limited commercial interest. However, the implementation of new funding mechanisms is facing several practical and financial challenges, such as a lack of expertise and guidelines, high transaction costs and difficulties to measure health outcomes. Furthermore, interviewees highlighted the need for increased collaboration and centralisation at a European and international level to make clinical research more efficient and reduce the need for additional funding.

**Conclusions:**

New funding mechanisms to support clinical research may become more important in the future but the unresolved issues identified in the current study warrant further exploration.

**Supplementary Information:**

The online version contains supplementary material available at 10.1186/s13063-021-05273-x.

## Background

Finding new therapeutic uses for existing medicines could lead to safe, affordable and timely treatment options for patients with high medical needs [1–3]. A major benefit of this strategy is that the pharmacokinetic, pharmacodynamic and toxicity profiles of approved medicines are well-known, so the new use can more easily be translated into phase II and III clinical trials [4–7]. In addition, approved medicines that have been on the market for several years are often relatively cheap compared to new medicinal products, especially if they are out of basic patent and regulatory protection and generic medicines exist. The wide availability and affordability of these medicines could facilitate clinical research and enable timely and affordable access for patients [8–10]. However, return on investment (ROI) for repurposed off-patent medicines is expected to be low or absent due to a lack of economic incentives [11, 12]. Pharmaceutical developers and their shareholders are therefore rarely interested to invest in repurposing opportunities for medicines that are out of basic patent and regulatory protection, essentially making these medicines ‘financial orphans’ [13, 14].

Due to this lack of commercial interest, new uses for approved, off-patent medicines are mainly studied in independent clinical trials initiated and led by researchers from academia, research institutes or collaborative groups [15, 16]. These trials are typically supported by public and philanthropic funds and aim to answer clinical questions that have an important impact on public health and patient needs but that are not addressed by industry-led trials [17, 18]. Synonyms include academic, non-commercial, physician-led, investigator-driven, investigator-initiated, investigator-sponsored or publicly funded clinical trials [19]. So far, researchers have been running numerous small proof-of-concept studies (i.e. phase I or II) to test the activity and safety of approved medicines in new therapeutic indications. The next step should be to confirm the results from exploratory trials in large confirmatory randomized controlled trials (RCTs) to avoid unproven off-label use of medicines based on low levels of clinical evidence [20]. However, confirmatory RCTs are expensive, time-consuming and labour-intensive. Moreover, limited and fragmented funding remains one of the most important barriers for initiating and completing these studies [21–24]. The average cost of a phase III clinical trial is difficult to establish as it depends on many factors and varies across therapeutic areas. In a study on pharmaceutical trials in the USA between 2004 and 2012, the cost of phase III trials ranged from US$11.5 million (dermatology) to US$52.9 million (pain and anaesthesia) [23, 25]. Even if we assume that an investigator-driven trial with approved medicines is less expensive than an industry-led trial with new medicines, for which the median cost was estimated at US$19 million [26], investments would still need to be substantial [27]. Therefore, additional financial support is needed to conduct robust, phase III clinical trials that address the translational gap in drug repurposing [6, 28].

The aim of this study was to identify and investigate potential mechanisms to fund independent clinical research with repurposed medicines by searching the scientific and grey literature. Moreover, we considered various perspectives on the application potential of the proposed funding mechanisms by conducting semi-structured interviews with European stakeholders.

## Methods

Literature was searched to identify and explore innovative models for organising and funding clinical drug repurposing research. Scientific literature was searched in MEDLINE (via PubMed) and Embase databases using search queries consisting of MeSH terms and key words in title and abstract (Supplementary material S1). Articles published until January 2021, in English, of which the full-text publication was available were selected. Moreover, additional literature was hand searched to clarify the structure, involved stakeholders, advantages, disadvantages and previous applications of the identified funding mechanisms for independent clinical research. Grey literature and publications from reference lists of the identified literature were also included.

Stakeholders with knowledge about clinical research, research funding mechanisms and/or drug repurposing were invited to participate in a semi-structured interview to identify new funding models and explore their application potential in Europe. Study participants were identified from author lists of scientific publications or grey literature and via the network of the research group and were selected via purposive sampling. Twenty-six people were contacted via e-mail and received an information sheet describing the objectives and design of the study. An interview guide was developed based on background information from scientific literature (Supplementary material S2). Questions related to the following topics: (i) the need for new finance models to support independent clinical research, (ii) interviewees’ experience with new finance models (i.e. public-private partnerships, social impact bonds, crowdfunding, other), (iii) stakeholders’ role in selected models, (iv) advantages, disadvantages and risks of selected models, and finally (v) the current and future role of new funding models for independent clinical research.

The interviews took place between November 2018 and February 2019. First, two pilot interviews were performed in the presence of three interviewers with a background in pharmaceutical and/or biomedical sciences to optimise the interview guide and to standardise the interview approach. The subsequent interviews were conducted in pairs or individually by the same interviewers, either face-to-face in the workplace of the participant or via phone or video call. The interviews were carried out in English or Dutch and lasted about 30 to 45 min each. All interviews were audio-recorded with informed consent from the study participants and transcribed ad verbatim and pseudonymized to protect participants’ personal information and ensure confidentiality. The interview transcripts, together with field notes, were analysed based on the framework analysis method by the first author of this study using the NVivo qualitative data analysis software [29, 30]. Some quotes used in this manuscript were translated from Dutch to English as accurately as possible to represent participants’ views.

The results of the literature review and the stakeholder interviews are collectively summarised in the “Results” section.

## Results

**Fig. 1:**
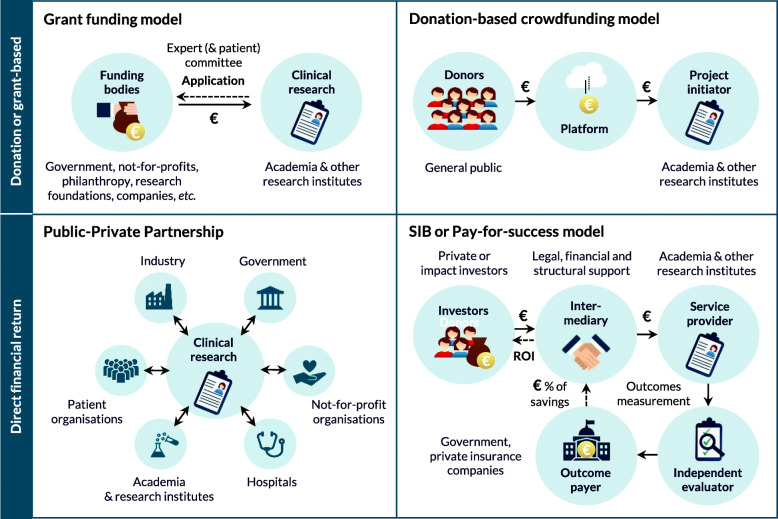
Overview of mechanisms to fund clinical drug repurposing research. SIB, social impact bond; ROI, return-on-investment

Based on the literature review, four potential mechanisms for funding independent clinical research with repurposed medicines were identified and are summarised in Fig. 1. Next, fourteen interviews were conducted with sixteen participants (two interviews involved two study participants simultaneously) to learn more about the application potential of such models in Europe. Interviewees represent various stakeholder groups, including not-for-profit or governmental organisations (*N* = 6), university hospitals and academia (*N* = 5), pharmaceutical industry (*N* = 2), a private bank (*N* = 1), a consultancy company (*N* = 1) and a health technology assessment body (*N* = 1) (Table 1).

**Table 1:** Characteristics of interview participants

Interview	Stakeholder group	Country
**A**	Academia	Ireland
**B**	University hospital	Belgium
**C**	Not-for-profit research organisation	The Netherlands
**D**	Consultancy and private bank (2 participants)	Belgium
**E**	Not-for-profit organisation	The UK
**F**	Not-for-profit organisation	Belgium
**G**	Health technology assessment body	Belgium
**H**	Not-for-profit research organisation	Belgium
**I**	University hospital	Belgium
**J**	Governmental funding organisation	The Netherlands
**K**	Academia	Belgium
**L**	Not-for-profit research organisation	Belgium
**M**	Academia	Belgium
**N**	Pharmaceutical industry (2 participants)	Belgium

### Grant or donation-based funding mechanisms

#### Traditional grant funding

The best-known mechanism to fund independent clinical trials is through grant funding programmes, which typically involve a funding body and numerous applicants (i.e. academia and research institutes). Funding can come from different sources, such as government agencies, not-for-profit and philanthropic organisations, universities, research foundations and pharmaceutical companies. In most cases, the research project should meet specific criteria to be eligible for the grant, and a project proposal has to be submitted for review by a committee of scientific experts and, sometimes, patients.

Even though grant funding is well established in all types of research, it has several limitations. Most importantly, grant funding programmes are highly competitive and the available funds are limited [31]. Funding applications for clinical trials with off-patent medicines in new therapeutic indications are often at a disadvantage because drug repurposing is not considered innovative. “Innovation in science and medicine is often measured by creation of something new, not by repurposing something old and available” [8]. However, Dr. Richard Thompson defended the innovative nature of drug repurposing as follows [32]: “Innovation is also equally about innovative ideas – finding new ways to deliver a service or improved ways to use current resources. Drug repurposing is an excellent example of this form of innovation: using a scientific approach to identify new uses for existing drugs”*.*

Not-for-profit organisations, government agencies and pharmaceutical companies are increasingly awarding grants specifically focused on clinical drug repurposing research in all disease areas [33–37] (Table 2). For example, the Anticancer Fund, a Belgian-based not-for-profit organisation, scientifically and financially supports independent clinical trials with off-patent repurposed medicines in cancer patients and has launched several calls for research proposals in this area [38]. CuresWithinReach and the Michael J. Fox Foundation, two US-based not-for-profit organisations, have also awarded multiple grants for investigating new therapeutic uses of existing medicines in various disease areas [39, 40]. Moreover, several government organisations, such as the Belgian Healthcare Knowledge Center (KCE), the Dutch ZonMw and the UK National Institute for Health Research (NIHR), have included drug repurposing as a focus area in their calls for funding of independent clinical research [41–43]. Pharmaceutical companies can also provide grants to support investigator-initiated clinical research with approved medicines, regardless of patent status. For example, Bayer ran a specific ‘Grants4Indications’ programme that provided grants and further financial support to explore new therapeutic indications for their own compounds [44].

**Table 2:** Funding opportunities for independent clinical repurposing research

Funding source	Organisation	Name of funding opportunity^a^	Available funds	Duration of research	Geographic area	Disease area
**Government organisations**	Belgian healthcare knowledge center (KCE) (BE)	KCE investigator-led trials	€10,000,000 per year, no defined max. amount per project	Results preferably within 5 years	International study possible under certain conditions	All
	ZonMw (NL)	Goed Gebruik Geneesmiddelen – Drug Rediscovery	Max. €1,000,000 per call	Not specified	International study possible if chief investigator and lead institution are NL-based	All
	National Institute for Health Research (NIHR) and Medical Research Council (MRC) (UK)	19/136 call for evaluating interventions for the diagnosis and treatment of autoimmune diseases	Case by case negotiations	Not specified	International study possible if chief investigator and lead institution are UK-based	Autoimmune diseases
**Companies**	Bayer	Grants4Indications	Case by case negotiations	Max. 2 years	International	All
**Not-for-profit organisations**	CuresWithinReach	ReGRoW Pilot	US$ 25,000–50,000 per project	12–36 months	Low and lower-middle-income-countries (LMICs)	Any unsolved disease in LMICs
	Michael J. Fox Foundation	Therapeutic Pipeline Program	US$2,000,000 per project	2–3 years	International	Parkinson’s disease
	The Anticancer Fund (ACF) and Rising Tide Foundation for Clinical Cancer Research (RTFCCR)	The RTFCCR/ACF Multi-arm Clinical Trial Award	US$ 3,000,000 in total	Not specified	International	Cancer

^a^ Non-exhaustive list of research calls with a focus on drug repurposing between January 2017 and January 2020

Interviewees highlighted the need for additional government funding to support independent research in all areas where market failure predominates due to a lack of incentives and ROI.

“The trick is of course to think of areas where things are not going well. [ … ] Only if there were a real market failure, you would have to look for other ways to finance this, through government funding in my case.” (Interview J)

Several interviewees also mentioned the increased need for a top-down or demand-oriented approach in which governments identify the most important unmet needs in healthcare and allocate research funding accordingly. A more active role of patient organisations in raising and allocating funds for independent research into treatment options addressing the highest patients’ needs was mentioned several times during the interviews. However, interviewees argued that not every patient organisation is equally well organised, and that not every disease is well represented, which is especially a problem for (ultra-)rare diseases.

Finally, some interviewees were concerned that a clinical research project that was funded with public money, once de-risked, may be taken over by a pharmaceutical company and end up in for-profit development.

“Traditional grant funding can work. [ … ] You have to rebuild a strong case based on the science for that and that can also deliver, but tends to end up reeling down to pharmaceutical pathway ultimately, so ends up generating the pharma profits.” (Interview E)

#### Crowdfunding

An alternative model to fund independent clinical research with repurposed medicines is by raising small donations from a large number of people via online platforms or portals, which is called crowdfunding (Table 3, Fig. 1). One of the major benefits of crowdfunding for clinical research is the opportunity to raise funds for innovative projects with a potentially high societal or patient impact but low commercial return, as is the case for repurposing off-patent medicines. The NeoART study is an example of a drug repurposing project that collected funds (£54,247) through a crowdfunding campaign on FutSci.com [46]. This phase II RCT aimed to investigate the efficacy of artesunate, an anti-malarial agent that was initially developed over 40 years ago, in colorectal cancer patients.

**Table 3:** Crowdfunding: basic principles

Crowdfunding can be either reward-based, equity-based or donation-based depending on the return that is offered to the funders [45]. Donation-based crowdfunding is most relevant to fund independent clinical research where financial ROI and other rewards are lacking. A donation-based crowdfunding model typically involves three types of stakeholders: the project initiator (in this case a research organisation seeking funding to conduct a clinical trial), the donors, and the online platform provider. Campaigns to fund clinical research can either be hosted on general-purpose (e.g. Indiegogo.com, Kickstarter.com) or research-focused crowdfunding platforms (e.g. Experiment.com, Consano.org). Each campaign features a description of the research project in lay language, a financial goal, and an indication of how close the campaign is to meeting this goal. Most campaigns specify a limited period to accept contributions. Some campaigns adhere to an ‘all-or-nothing’ or ‘fixed-funding’ model, meaning that donations are kept only if the financial goal is met or exceeded.	

However, interviewees in this study raised some practical limitations and ethical concerns regarding crowdfunding for clinical research. First, setting up a successful crowdfunding campaign can be time-consuming and challenging as it requires a lot of strategic planning and a multidisciplinary support team. High overhead and administrative costs, including transaction costs of platforms, can make crowdfunding efforts less efficient. One interviewee, having had experience with setting up crowdfunding campaigns to support repurposing research, confirmed this challenge.

“We’ve run a few crowdfunding campaigns ourselves [ … ] they are lots of hard work for limited success.” (Interview E).

All interviewees agreed that large clinical trials are too expensive to fund via a crowdfunding approach. Still, it could be used to de-risk early-stage projects and thus increase their chance of success in obtaining traditional research grants.

“Clinical trials are expensive, so getting that amount in a crowdfunding effort is close to impossible.” (Interview H)

Importantly, research that receives the most funds via crowdfunding may not always address the highest unmet medical needs. In fact, two interviewees pointed out that clinical research into rare diseases is at a disadvantage in crowdfunding campaigns since fewer people have an emotional connection to such diseases.

“Because conditions are rare, there isn’t a huge public understanding of many of the conditions and probably not a huge public understanding of what is needed to deliver research either, so I think that makes it a challenging route and certainly not sustainable one.” (Interview E)

### Mechanisms with a direct financial return

#### Public-private partnerships

A public-private partnership (PPP) is a collaboration between at least one public partner and one private partner with a common goal, for example improving health outcomes. PPPs are no longer a new concept in the healthcare sector and have been established to serve many different purposes [47, 48]. Some PPPs tackle specific precompetitive topics, while others focus more on development or access to medicines. The structure of each PPP may vary depending on the involved stakeholders, such as the pharmaceutical industry, academia, government, not-for-profit organisations, hospitals, research and patient organisations (Fig. 1). Multi-stakeholder PPPs allow synergies and sharing of knowledge, expertise and resources between all partners. A PPP can be seen as a win-win model that aims to reduce development costs, to increase the scale and scope of the research, and to share the financial risks of drug development between all partners [27]. Consequently, PPPs have been proposed as a potential model to facilitate and fund drug repurposing research [49, 50].

Indeed, various PPPs have been established between academic researchers, public funders and the pharmaceutical industry to support drug repurposing research, but most are situated in the precompetitive space and focus on the repurposing of shelved compounds that initially failed in clinical studies for another indication and were never marketed. Examples include the UK Medical Research Council (MRC) Mechanisms for Human Diseases Initiative, the US National Center for Advancing Translational Sciences (NCATS) Discovering New Therapeutic Uses for Existing Molecules initiative, the US Clinical and Translational Science Awards (CTSA) Pharmaceutical assets Portal and the EU Innovative Medicines Initiative (IMI) pilot programme on a clinical compound bank for repurposing [51, 52]. In the product development area, there is one US-based PPP between the Therapeutics for Rare and Neglected Diseases programme of the US National Institutes of Health (US NIH) Chemical Genomics Center, The Leukemia & Lymphoma Society, and University of Kansas Cancer Center, which is called The Learning Collaborative. This partnership repurposed auranofin, an off-patent medicine initially approved to treat rheumatoid arthritis in the mid-1980s, for the treatment for relapsed chronic lymphocytic leukaemia [49]. Moreover, the UK-based aPODD foundation is open to supporting partnerships in drug repurposing projects for paediatric oncology indications [53] and the Dutch Fair Medicine foundation proposes a coalition model between patient associations, hospitals, researchers, health insurers, large and small investors and pharmaceutical developers to develop sustainable and affordable medicines, including repurposed medicines [36].

Despite the many potential benefits of PPPs, interviewees argued that they do not offer a sustainable solution for off-patent drug repurposing due to the lack of incentives for the private partners. Social corporate responsibility was mentioned as a potential reason for companies to participate in such a PPP, but this was not deemed as sufficiently motivating, unless in areas where the competitive pressure is low, for example for finding new treatment options for neglected diseases in low- and middle-income countries (LMICs).

#### Social impact bonds or pay-for-success models

A social impact bond (SIB) is an innovative model that leverages private investments to develop public health services or interventions. A SIB, also referred to as pay-for-success financing, is a formal agreement between an outcome payer (typically a government, payer or private insurance company) and a service provider (in this case a not-for-profit or research organisation seeking funding to conduct one or more clinical trials), where the outcome payer specifies a desired outcome and guarantees to pay back the investors their upfront investments plus a return if this outcome is reached (Table 4, Fig. 1). So far, SIBs have predominantly been applied to fund preventive health measures that could result in significant long-term health care savings [59, 60]. The UK-based organisation Findacure started exploring a SIB model to incentivise investment into drug repurposing clinical trials in rare diseases, in collaboration with various organisations including CuresWithinReach, Mission:Cure, Numbers4Good and Costello Medical [34, 36, 60–62]. More specifically, the goal of this Rare Disease Drug Repurposing SIB is to create a portfolio of up to ten phase II efficacy clinical trials that, if successful, could lead to off-label prescription of affordable repurposed medicines for patients with rare diseases who currently have no treatment. The improved outcomes and reduced care needs of those patients would then result in significant savings for healthcare systems and a proportion of these savings would subsequently be paid back by the outcome payer (in this example, the UK National Health Service) to the investors as a success payment [9]. Recently, the US-based think tank Helena and its partners proposed a similar financial model to fund off-patent drug repurposing for Alzheimer’s disease [63].

**Table 4:** Impact investing: basic principles

Currently, about 441 million dollars have been raised for 138 Social Impact Bonds (SIBs) worldwide [54]. The use of the term ‘bond’, which refers to a fixed income instrument in finance circles, is somewhat misleading because the investors’ return in a SIB is dependent on the success of achieving predefined outcomes [55]. In fact, a SIB is more similar to a public-private partnership between private or impact investors, a service provider and an outcome payer (Fig. 1) [56]. Most SIBs include an intermediary to convene all stakeholders and provide legal, financial and structural support. An independent evaluator typically measures the outcomes, which are key to determine the cost savings, success payments and social impact of a project. For a SIB to be successful, outcomes should be quantifiable and should lead to clear societal and government savings. The SIB model should not be confused with another upcoming finance model, which is called ‘venture philanthropy’. The venture philanthropy model is based on a partnership between a charity and a drug company and provides a mechanism for not-for-profit organisations to help finance the development of a treatment in return for a share in profits, which can later be reinvested in other new treatments [36]. For example, the Cystic Fibrosis Foundation invested US$150 million in Vertex Pharmaceuticals for the development of ivacaftor, and had a return of US$3.3 billion in exchange for its royalty interests [57]. Even though this model may lead to promising new treatments, ethical questions have been raised about the sustainability of a model that maximises profits using philanthropic funds [58].	

SIBs are a relatively new way to fund health programmes, so evidence with regard to their efficacy to support clinical research with repurposed medicines is limited. Accordingly, only few interviewees had experience with SIBs, although everyone was open to the idea and recognised their potential value for the repurposing of off-patent medicines. Still, interviewees described several difficulties and potential drawbacks of SIBs. First, not every not-for-profit programme is fit for a SIB. SIBs need easily quantifiable outcomes that can be achieved in a limited time period and lead to clear government savings [64]. Interviewees voiced some concerns about the identification of robust clinical outcome measures to demonstrate social impact and cost savings of a new treatment and about the long duration and low success rates of most clinical trials.

“There is actually a big risk to those organisations [service providers] in getting involved if they haven’t set up the measure of success well or they’ve been over ambitious in what they’re saying they can achieve and don’t deliver. They won’t receive the returns they need to pay their costs.” (Interview E)

Interviewees who had experience with SIBs emphasised the difficulty of securing commitment and resources from governments, especially in multi-level governance and multi-payer systems.

“It will not be a problem to find private investments. [ … ] I think the bottleneck is in the public funds.” (Interview D)

Finally, statistical, legal and contracting expertise is required for establishing a SIB, and the transaction costs and organisational burden are high. Therefore, sufficient start-up funding is needed. One interviewee was of the opinion that governments should provide administrative, legal and financial support for setting up SIBs that aim to achieve better social and health outcomes. If SIBs were to become more common, transaction costs would automatically decrease as a result of the standardisation of legal forms and contracts.

“I think given that charities and third sector organisations are generally those organisations that are going to deliver these interventions, they don’t have a huge amount of disposable income to put all of that work and infrastructure in place.” (Interview E)

Overall, interviewees believed that the potential benefits of SIBs outweigh their costs and risks and that their application potential at a national and international level warrants further exploration to support research into new uses for existing medicines.

### Improving efficiency of independent clinical research

Interviewees highlighted that, in addition to exploring new funding mechanisms, independent clinical research should become more efficient. Even though parallelism in research may increase productivity to some extent, there is a lot of fragmentation and duplication of research efforts. Moreover, independent clinical trials are often not sufficiently powered to show evidence of clinical efficacy, probably also due to the limited funds and less organisational support compared to industry-sponsored trials.

“I am not saying that it’s always the case, but it is a personal opinion that there is probably too much fragmentation to be very efficient.” (Interview N)

Increased national and international cooperation and consortium-building between research groups and foundations could be key to address this problem. Furthermore, interviewees mentioned that funding efforts to support clinical research, such as grant-funding programmes and SIBs, should be organized at a European or international level to become more feasible and efficient.

“You have to organise [research funding] on an international level to reach critical mass, that is just a given.” (Interview K)

Yet, harmonisation and centralisation of independent clinical research on a European level would require the establishment of one or more coordinating centres or, as suggested by one of the interviewees, a multi-stakeholder review board or steering committee overviewing independent clinical trials in Europe. The European Organisation for Research and Treatment of Cancer (EORTC) was put forward several times as an ideal candidate to fulfil such a role within cancer research.

“We see a third partner to guide the process and to make sure that it is useful, that it is done in a correct way and that you can also make connections with European funds or with other research institutes in other countries.” (Interview N)

RCTs are still the golden standard for determining the efficacy of a medicine in a new therapeutic indication but they entail high costs, a long duration and a substantial administrative burden [65]. To address these challenges, interviewees mentioned the need for optimising clinical trial designs and methodology for drug repurposing research.

“What is important to us to consider first, is an optimization of the methodology of the trial to be able to use other designs, other methodology, other technology that can limit the need for financing or the costs, if I may say, for the trial.” (Interview N)

## Discussion

This explorative study aimed to address a key financial challenge in drug repurposing, which is to find sufficient funding to conduct robust, phase III clinical trials with approved off-patent medicines. Even though the costs of repurposing an existing medicine are said to be lower than for developing a de novo compound, they are still relatively high and the development carries a lot of risk [5, 66]. Yet, revenues for off-patent medicines are not expected to increase substantially after adding a new therapeutic indication since payers are unlikely to agree to pay a higher price for an existing medicine, which is often already prescribed off-label for the new indication. Moreover, new uses for off-patent medicines are particularly difficult to protect from generic competition [10]. As a result, pharmaceutical companies rarely pursue new indications after expiry of basic patent and/or regulatory protection, especially for inexpensive small-molecule medicines [12].

Researchers from academia, government and other research institutes conduct many small proof-of-concept studies to test repurposing hypotheses, but often lack the funding to confirm their results in expensive confirmatory trials [20]. Furthermore, resources are needed to reduce the administrative burden on clinical investigators when initiating or engaging in non-commercial clinical research (e.g. administrative support for protocol development, ethics approval, etc.). Additional funding is also needed to facilitate clinical adoption of treatments once the trials are completed (i.e. via regulatory approval and reimbursement procedures) [10, 67]. Involved stakeholders have proposed various funding mechanisms to support independent clinical research with repurposed medicines, ranging from traditional grant funding programmes to highly innovative SIB models. In addition to outlining the theoretical aspects, we considered various perspectives on the potential value of such funding mechanisms in Europe through semi-structured interviews with experts in this field. Several key learnings about the future perspectives of the proposed funding mechanisms can be derived from this study.

Traditional grant funding by government and philanthropic organisations is still the main driver to support independent clinical trials [33]. With investments of more than US$40 billion a year, the US NIH is the largest public funder of biomedical research in the world [68]. In Europe, the European Commission is supporting multinational research through its Horizon Research and Innovation programmes, the last framework programme ‘Horizon 2020’ provided about €80 billion of funding over 7 years (2014–2020) [69]. Moreover, numerous national funders are investing in independent clinical research, for example the UK NIHR and MRC, the German Research Foundation, the French Institut national de la santé et de la recherche médicale (INSERM), the Innovation Fund Denmark and many more. However, all these programmes are extremely competitive and have relatively low acceptance rates [22]. Interviewees highlighted the importance of public funding in all research areas where market failure predominates. However, they recognised that public funds are limited and should therefore be allocated to research that addresses the highest unmet needs in healthcare, preferably in consultation with patient organisations and society at large, for example via citizens workshops and questionnaires measuring societal preferences.

Crowdfunding was discussed as an alternative way to raise funds. This model enables patient and public engagement in prioritizing clinical research goals and increases public awareness of research needs [70, 71]. Crowdfunding can be particularly interesting for early-career investigators, who generally have a lower chance of success in competitive grant programmes [72, 73]. However, previous research suggests that while crowdfunding could be a viable model to support small proof-of-concept trials, it would not be sufficient to fund large and more expensive RCTs [45, 73]. Interviewees confirmed that the amount of funding required to support large RCTs with repurposed medicines would likely surpass the willingness to pay of ‘the crowd’. Indeed, the success of a crowdfunding campaign is not guaranteed. For example, in 2015, Sharma et al. identified twenty campaigns for clinical research (not focused on repurposing research), of which seven were still ongoing. Of the thirteen completed campaigns, only eight (62%) reached their financial goal. The funds raised in these campaigns ranged from US$3600 to about US$3 million, with an average of US$540,000 and a median of US$167,000 [74, 75]. Moreover, an inconclusive or negative trial outcome could erode public trust [76]. Moreover, previous research argued that research funding should be based on a project’s scientific merit rather than its potential to attract emotional donations [70, 76–79]. Additional ethical concerns of crowdfunded research, which were not mentioned by the interviewees, include a lack of control over the quality, scientific integrity and feasibility of crowdfunded research [70, 72, 76, 78].

Another model that has been successful in numerous areas of drug development is the multi-stakeholder PPP. Even though several precompetitive PPPs have been established to identify and develop repurposing opportunities for shelved experimental assets, interviewees were of the opinion that this model would not be viable to fully fund clinical research to repurpose off-patent, generic or biosimilar medicines due to a lack of financial incentives for private stakeholders. Still, lessons can be learned from successful international product development partnerships like the Drugs for Neglected Diseases initiative (DNDi) and the Medicines for Malaria Venture (MMV), both well-established global PPPs that leverage companies’ social corporate responsibility objectives to achieve their goal and typically operate in areas with low competitive pressure [27, 80]. In fact, these PPPs already included several rescued and repurposed medicines in their research portfolios [27, 81, 82].

A final model that was explored in this study is the pay-for-success or social impact bond (SIB) model that leverages private investments to develop public health services or interventions. Only few interviewees had prior knowledge of the SIB model, but they were of the opinion that it warrants further exploration. A SIB concept can be described as a win-win-win model that, if successful, improves health outcomes, reduces healthcare spending and realises economic return [83]. Additionally, SIBs enable a shift in financial risk from governments to investors compared to the grant funding model, attract new sources of capital to scale up health programmes and research and stimulate not-for-profit organisations and researchers to focus on productivity and outcomes [59]. SIBs could also be scaled-up to an international level to share the risks among more investors and distribute the pay-outs between outcome payers [61]. Still, several difficulties and potential drawbacks of SIBs have been reported in literature [64], which were echoed by interviewees in this study. One critical unresolved issue is the difficulty to measure the social impact and predict the cost savings that could be delivered by using repurposed medicines in clinical practice. Experience in measuring these outcomes may be gained from pay-for-performance or outcome-based managed entry agreements that are increasingly being used for market access of high-cost innovative medicines in Europe [84, 85]. In addition, a SIB would require initial public or philanthropic investments to cover the implementation and transaction costs. Besides, establishing a SIB requires a long-term vision and the political will of governments, payers and/or insurance companies to guarantee success payments for projects that will only pay off in a couple of years [9, 86]. While those challenges should definitely be considered, interviewees were of the opinion that it should not prevent this model from being tested in a pilot project.

In addition to identifying new funding mechanisms, interviewees expressed the need to enhance collaboration and centralisation at a European level to make clinical research more efficient and maximise the value of limited public funding. This finding is in line with emerging recommendations from the scientific community to increase international clinical trial collaboration in multiple disease areas, particularly also the collaboration between high-income countries (HICs) and LMICs [87–91]. Despite the clear benefits for increased cross-border collaboration in clinical research, only about 3% of academic trials are multinational, compared to 30% of industry-led trials [92]. Inadequate funding mechanisms, like national grants with geographic restrictions, and mismatches in international clinical regulations and guidelines may stifle multinational independent research [21, 22]. Several multinational funding programmes have been set up to overcome this challenge. One example is the European and Developing Countries Clinical Trial Partnership (EDCTP), which is focused on finding solutions for HIV/AIDS, tuberculosis and malaria as well as other poverty-related infectious diseases in sub-Saharan Africa. The EDCTP combines investments from the European Commission, national member countries and other international partners [93]. Another example is the Nordic Trial Alliance that aims to enhance Nordic cooperation on clinical multi-centre trials and is funded by the Nordic Council of Ministers and NordForsk [94]. Moreover, European countries could join forces in transnational research and innovation projects via a European Research Area Network (ERA-Net) co-fund scheme [22, 95]. Other collaborative initiatives such as the US Clinical Trials Transformation Initiative (CTTI), the European Clinical Research Infrastructure Network (ECRIN) or multinational disease-specific research organisations (e.g. EORTC) could potentially function as coordinating centres and facilitate patient-centred approaches for increasing the quality and efficiency of international clinical trials. Furthermore, interviewees mentioned that advancements in research methodology and technology could lead to more innovative trial designs for drug repurposing research [96–98]. Various study designs have been proposed in scientific literature to replace or at least complement traditional RCTs, such as pragmatic and low-interventional trials, registry-based RCTs [99, 100], N-of-1 trials for rare diseases [101], multi-arm/multi-stage or platform trials [96–98] and real-world patient data studies [8, 102]. Yet, further research focused on clinical research methodology is needed to explore the application potential of such study designs to drug repurposing.

Over the past months, the global COVID-19 pandemic highlighted the significant potential of repurposed medicines to help tackle urgent global health threats in a timely and affordable manner. Numerous regional, national and international clinical trials investigating a wide-array of repurposing candidates have been initiated at an unprecedented rate [103]. However, questions have been raised about whether conducting that many ‘small’ clinical trials is a good use of resources [104]. Various stakeholders have expressed concerns about the risk of duplication of research, the competition for patients and research funds, the ethical issue of enrolling so many patients in individual control groups and the ability of these trials to support robust regulatory and treatment decision-making [105]. Indeed, this pandemic emphasised the need for better coordination in clinical research and funding at the international level [105], which was also highlighted by the current study. The EU ‘ERAvsCorona’ action plan was launched to provide rapid dedicated funding and infrastructure to support large, EU-wide clinical trials for the management of coronavirus patients. In addition, key stakeholders underscored the value of adaptive platform trials for accelerating the identification of effective COVID-19 treatments [105, 106]. Examples of such trials investigating repurposed drugs for COVID-19 include the international ‘Solidarity’ trial launched by the WHO and the UK-based ‘RECOVERY’ trial led by the University of Oxford. It is apparent that, when rapid action is needed, the financial constraints that typically impede repurposing research with off-patent medicines can be largely removed [107, 108]. Yet, it remains to be seen whether the lessons learned from this pandemic will be translated to the repurposing of medicines in other disease areas in the future.

The current study is exploratory in nature and has three main limitations. The first limitation is the small number of interview participants representing many different stakeholder groups. To learn valuable insights from this small study sample and reach the point of data saturation, only stakeholders with profound expertise in either drug repurposing, clinical research or funding mechanisms were included, and the results were complemented with information from the scientific literature as much as possible. The multi-stakeholder approach enabled us to capture diverse opinions about the application potential of the proposed funding mechanisms. A second limitation is the fact that the majority of interview participants are based in Belgium. Nevertheless, more than half of the participants had many years of experience of working in a European or international organisation and context, which is why the results can be extrapolated to the European level. Third, this study applied qualitative research methods, so our results do not allow us to quantify the stakeholders’ perspectives or opinions about proposed funding mechanisms. If a new funding mechanism were to be tested in a pilot project, it could be useful to incorporate a quantitative study, for example a survey, involving different stakeholder groups to measure and evaluate the advantages and disadvantages of the studied mechanisms in practice.

## Conclusion

At present, there is a lot of encouraging science to support the repurposing of medicines in various disease areas, but the current pharmaceutical model is not designed to accommodate the development of medicines for which commercial prospects are low. This study highlighted the need to enable and promote independent clinical research in all areas where market failure predominates, including the repurposing of off-patent medicines, and clarified several ways in which this could be achieved.

First, additional public funding could be provided to conduct independent clinical trials with repurposed medicines, especially confirmatory phase III RCTs. Given that clinical trials are expensive and have a high risk of failure, it is important to adopt a robust method for allocating the limited public funds to the research projects with the highest potential benefit to patients and society.

Second, while public funding is indispensable to support independent clinical research, the feasibility of new funding mechanisms, such as SIBs or PPPs, could be explored further in one or more pilot projects.

Finally, there is a clear need for increased harmonisation and centralisation of clinical research and funding at the European and international level in order to reduce fragmentation and maximise the value of limited resources.

## Supplementary Information

**Additional file 1: S1.** Literature search.

**Additional file 2: S2.** Interview topic guide.

## Data Availability

The raw data supporting the conclusions of the current study can be made available by the corresponding author to any qualified researcher on reasonable request.

## Abbreviations

CTSA: Clinical and Translational Science Awards
CTTI: Clinical Trials Transformation Initiative
DNDi: Drugs for Neglected Diseases initiative
ECRIN: European Clinical Research Infrastructure Network
EDCTP: European and Developing Countries Clinical Trial Partnership
EORTC: European Organisation for Research and Treatment of Cancer
ERA-Net: European Research Area Network
HICs: High-income countries
IMI: Innovative Medicines Initiative
IP: Intellectual property
KCE: Belgian Healthcare Knowledge Center
LMICs: Low- and middle-income countries
MMV: Medicines for Malaria Venture
MRC: Medical Research Council
NCATS: National Center for Advancing Translational Sciences
NIHR: National Institute for Health Research
PPP: Public-private partnership
RCT: Randomized controlled trial
ROI: Return-on-investment
SIB: Social impact bond
US NIH: United States National Institutes of Health
