# Supplementary material for: Exploring new uses for existing drugs: innovative mechanisms to fund independent clinical research
Source: Trials. 2021 May 4;22:322. doi: 10.1186/s13063-021-05273-x (PMC8093905; doi:10.1186/s13063-021-05273-x)
Supplement: Supplementary file 1 — Additional file 1: S1. Literature search. [file 13063_2021_5273_MOESM1_ESM.docx]

**S1. LITERATURE SEARCH**

**MEDLINE search (February 15, 2021)**

(("Drug Repositioning"[Mesh] OR "Repurposed drug*"[tiab] OR "Repositioned drug*"[tiab] OR "Drug repurposing"[tiab] OR "Drug repositioning"[tiab] OR "Drug reprofiling"[tiab] OR "Drug redirecting"[tiab] OR "Drug rediscovery"[tiab] OR "Drug retasking"[tiab]) OR (("Pharmaceutical Preparations"[Mesh] OR "Drug*"[tiab] OR "Medicine*"[tiab] OR "Medication*"[tiab] OR "Medicinal product*"[tiab] OR "Pharmaceutical product*"[tiab] OR "Medicament*"[tiab]) AND ("Repositioning"[tiab] OR "Repurposing"[tiab] OR "Rediscovery"[tiab] OR "Redirecting"[tiab] OR "Reprofiling"[tiab] OR "Retasking"[tiab] OR "Re-profiling"[tiab] OR "Re-tasking"[tiab]))) AND ("Fund*" [tiab] OR "Financ*" [tiab]) NOT fundamental

**Criteria:** Articles published until January 2021, in English, of which the full-text publication was available

**Embase search (February 15, 2021)**

('Repurposed drug*':ti,ab OR 'Repositioned drug*':ti,ab OR 'Drug repurposing':ti,ab OR 'drug repositioning'/exp OR 'Drug repositioning':ti,ab OR 'Drug reprofiling':ti,ab OR 'Drug redirecting':ti,ab OR 'Drug rediscovery':ti,ab OR 'Drug retasking':ti,ab) OR (('Drug*'/exp OR 'Drug*':ti,ab OR 'Medicine*':ti,ab OR 'Medication*':ti,ab OR 'Medicinal product*':ti,ab OR 'Pharmaceutical product*':ti,ab OR 'Medicament*':ti,ab) AND ('Repositioning':ti,ab OR 'Repurposing':ti,ab OR 'Rediscovery':ti,ab OR 'Redirecting':ti,ab OR 'Reprofiling':ti,ab OR 'Retasking':ti,ab OR 'Re-profiling':ti,ab OR 'Re-tasking':ti,ab)) AND ('fund*':ti,ab OR 'financ*':ti,ab) NOT fundamental

**Criteria:** Articles published until January 2021, in English, of which the full-text publication was available

**Article selection and inclusion**

| # Articles | Database |
| --- | --- |
| Total | MEDLINE: 149  Embase: 116 |
| After title & abstract screening | MEDLINE: 50  Embase: 14 |
| After full-text screening | MEDLINE: 19  Embase: 2 |
