# Supplementary material for: Exploring new uses for existing drugs: innovative mechanisms to fund independent clinical research
Source: Trials. 2021 May 4;22:322. doi: 10.1186/s13063-021-05273-x (PMC8093905; doi:10.1186/s13063-021-05273-x)
Supplement: Supplementary file 2 — Additional file 2: S2. Interview topic guide. [file 13063_2021_5273_MOESM2_ESM.docx]

**S2. INTERVIEW TOPIC GUIDE**

| THEME | QUESTION(S)* |
| --- | --- |
| Introduction | 1. Could you please elucidate your [*professional background/field of research*]? |
| Need | 1. In your opinion, is there a need for new finance models to support non-commercial/independent clinical research, such as clinical trials with off-patent repurposed drugs? 2. Who should be responsible for fundraising and allocating funds for non-commercial scientific projects? |
| Knowledge/  Experience | 1. Could you name one or more existing social finance models that may be used for financing clinical research? 2. What is your experience with [*PPPs/SIBs/crowdfunding/other*]? |
| Stakeholders | 1. Who are the main stakeholders involved in [*PPPs/SIBs/crowdfunding/ other*]? 2. Is there a need to involve additional stakeholders? If yes, who? |
| Advantages, disadvantages and risks | 1. What are the advantages of social finance models?  - More specifically of [*PPPs/SIBs/crowdfunding/other*]?  1. What are the disadvantages and risks of social finance models?  - More specifically of [*PPPs/SIBs/crowdfunding/other*]?  1. In your opinion, do the advantages outweigh the disadvantages and risks? |
| Current role | 1. What is the current role of these finance models in Belgium/Europe?  - Can you name any examples of social finance models used in practice, preferably in the health care sector? |
| Future perspectives | 1. What needs to change in order to optimize the implementation of social finance models, more specifically [*PPPs/SIBs/crowdfunding/other*], in Belgium/Europe? |

** Information between square brackets was adapted depending on area of expertise of each interviewee.*
